# Host inflammatory response is the major factor in the progression of *Chlamydia psittaci* pneumonia

**DOI:** 10.3389/fimmu.2022.929213

**Published:** 2022-09-02

**Authors:** Zhenjie Zhang, Peihan Wang, Chuanmin Ma, Jing Wang, Wenxin Li, Chuansong Quan, Huae Cao, Hongfeng Guo, Liang Wang, Chengxin Yan, Michael J. Carr, Ling Meng, Weifeng Shi

**Affiliations:** ^1^ Key Laboratory of Etiology and Epidemiology of Emerging Infectious Diseases in Universities of Shandong, Shandong First Medical University and Shandong Academy of Medical Sciences, Taian, China; ^2^ Beijing Institute of Genomics, Chinese Academy of Sciences, China National Center for Bioinformation, Beijing, China; ^3^ Department of Respiratory and Critical Care Medicine, Affiliated Hospital of Jining Medical University, Jining, China; ^4^ Department of Infectious Disease, Xintai Third People’s Hospital, Xintai, China; ^5^ Department of Medical Imaging, The Second Affiliated Hospital of Shandong First Medical University, Taian, China; ^6^ National Virus Reference Laboratory, School of Medicine, University College Dublin, Dublin, Ireland; ^7^ International Collaboration Unit, International Institute for Zoonosis Control, Hokkaido University, Sapporo, Japan; ^8^ Department of Respiratory and Critical Care Medicine, The Second Affiliated Hospital of Shandong First Medical University, Taian, China; ^9^ School of Public Health, Shandong First Medical University and Shandong Academy of Medical Sciences, Taian, China

**Keywords:** *Chlamydia psittaci*, community-acquired pneumonia, transcriptomic profiling, inflammatory response, inflammatory cytokines

## Abstract

**Purpose:**

*Chlamydia psittaci* (*C. psittaci*) has caused sporadic, but recurring, fatal community-acquired pneumonia outbreaks worldwide, posing a serious threat to public health. Our understanding of host inflammatory responses to *C. psittaci* is limited, and many bronchitis cases of psittaci have rapidly progressed to pneumonia with deterioration.

**Methods:**

To clarify the host inflammatory response in psittacosis, we analyzed clinical parameters, and compared transcriptomic data, concentrations of plasma cytokines/chemokines, and changes of immune cell populations in 17 laboratory-confirmed psittacosis cases, namely, 8 pneumonia and 9 bronchitis individuals, in order to assess transcriptomic profiles and pro-inflammatory responses.

**Results:**

Psittacosis cases with pneumonia were found to have abnormal routine blood indices, liver damage, and unilateral pulmonary high-attenuation consolidation. Transcriptome sequencing revealed markedly elevated expression of several pro-inflammatory genes, especially interleukins and chemokines. A multiplex-biometric immunoassay showed that pneumonia cases had higher levels of serum cytokines (G-CSF, IL-2, IL-6, IL-10, IL-18, IP-10, MCP-3, and TNF-α) than bronchitis cases. Increases in activated neutrophils and decreases in the number of lymphocytes were also observed in pneumonia cases.

**Conclusion:**

We identified a number of plasma biomarkers distinct to *C. psittaci* pneumonia and a variety of cytokines elevated with immunopathogenic potential likely inducing an inflammatory milieu and acceleration of the disease progression of psittaci pneumonia. This enhances our understanding of inflammatory responses and changes in vascular endothelial markers in psittacosis with heterogeneous symptoms and should prove helpful for developing both preventative and therapeutic strategies.

## Introduction

*Chlamydia psittaci* (*C. psittaci*) is an obligate intracellular bacterium. Human routes of zoonotic infection are through inhalation of *C. psittaci*-containing secretions from dried bird feces (1). Recently, sporadic psittacosis has been reported in numerous regions, including China, the United States, and Europe (2–5). Typical clinical signs include fever, cough, headache, and dyspnea—classic symptomatology of atypical pneumonia (6, 7). *C. psittaci* is more pathogenic and replicates more rapidly than other chlamydia (8, 9), and can have a disseminated, severe clinical course with disease manifestations in organs outside the respiratory tract (10). Therefore, psittacosis poses a public health risk warranting a deeper understanding of the underlying pathophysiology.

Previous studies on respiratory viruses (influenza A, SARS-CoV, and SARS-CoV-2) have shown that viral infections can induce hypercytokinemia, leading to uncontrolled pro-inflammatory responses elicited by the infecting pathogen (11–13). Immune disorders caused by hypercytokinemia are usually closely correlated with the occurrence and progression of severe pneumonia (12). Little is known of the effects of *C. psittaci* infection on the host transcriptome and how hyperactive immune responses are activated, mainly due to the relative infrequency of *C. psittaci* infection and lack of well-documented human-to-human transmission.

In late 2020, we reported a large-scale outbreak of human-to-human transmission in Shandong Province, China, with 22 confirmed cases, eight of which rapidly progressed to severe pneumonia, leading to one death (14). To identify and characterize the host inflammatory response to *C. psittaci* infection, we collected peripheral blood samples from these psittacosis cases, as well as healthy controls, and performed transcriptome profiling and also functional analyses. We have characterized distinct transcriptomic and cytokine signatures of the host inflammatory responses in psittacosis with bronchitis and pneumonia.

## Materials and methods

### Ethical approval

This study was approved by the ethics committee of the Second Affiliated Hospital of Shandong First Medical University on 1 December 2020 (No. 2020-077). The study followed the principles of the Declaration of Helsinki and the standards of Good Clinical Practice as defined by the International Conference on Harmonization (https://www.ich.org). A written informed consent was obtained from each *C. psittaci* case or their guardians. The research-related information was used anonymously.

### Clinical data and sample preparation

**Figure 1:**
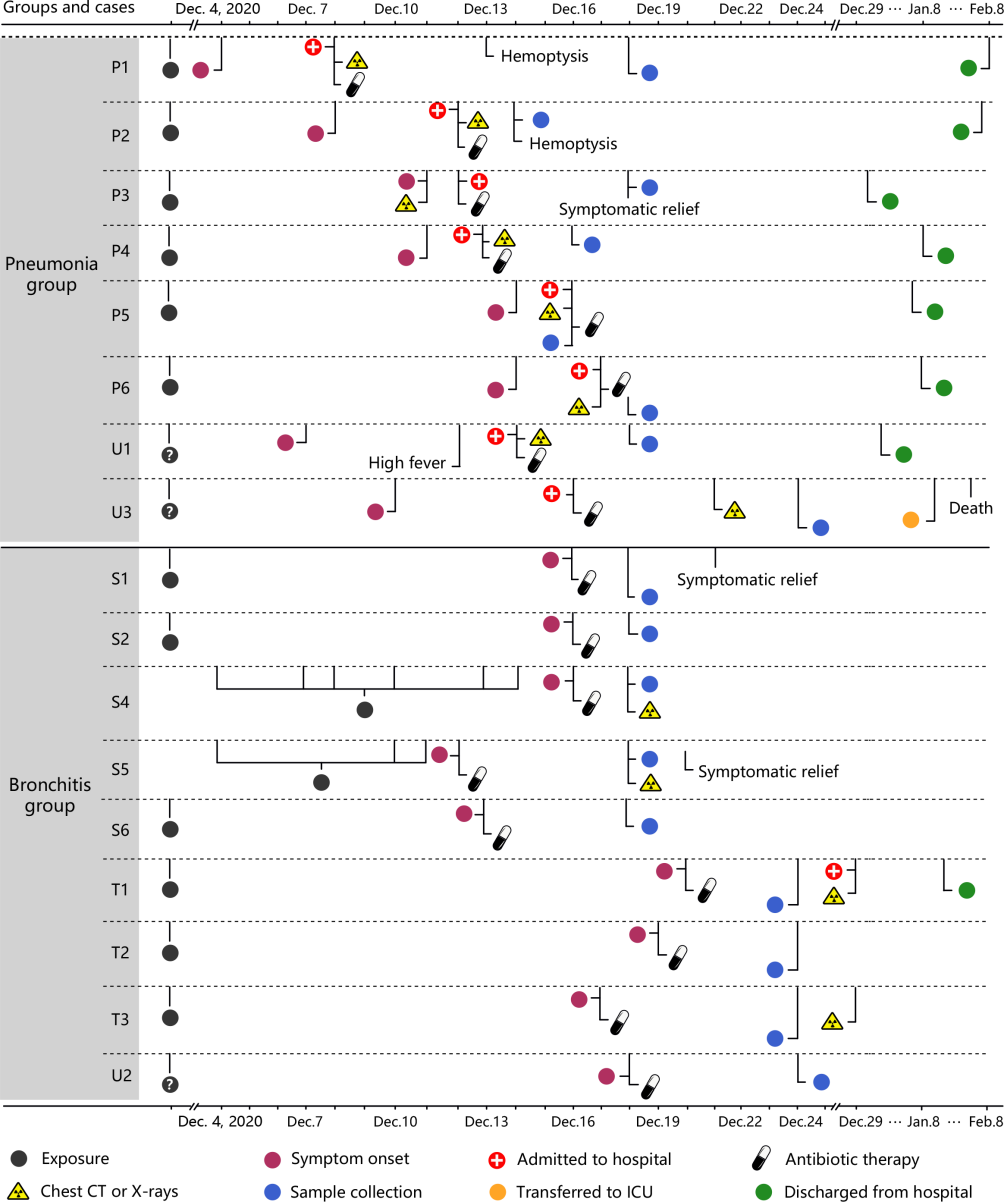
Timelines of the 17 *C. psittaci* cases. Each timeline represents one confirmed case from symptom onset to discharge from hospital. CPS: *Chlamydia psittaci*. P represents patients with primary infections. U represents patients with an unknown source of infection. S represents cases with secondary infections of *C. psittaci*. T represents third-generation cases.

Between 4 December and 29 December 2020, participants included 17 laboratory-confirmed *C. psittaci* cases, excluding 5 asymptomatic cases (14). Clinical information, including complete blood counts, chest computed tomography (CT) imaging, X-rays, and blood biochemistry, of the *C. psittaci* cases were collected at the earliest time points after hospitalization. They were classified into psittacosis pneumonia and bronchitis groups according to the infection location or severity of the infection, such as the presence or absence of invasive changes in the lungs shown on CT or X-ray. According to the grouping criteria, the five asymptomatic cases were excluded, and were not considered as an individual group due to their small sample size. Blood, nasopharyngeal swabs, and sputum from each case were collected at the same time point (“Sample collection” in **Figure 1**). Whole blood samples were stored in vacutainers (Becton Dickinson, New Jersey, USA) to separate plasma. Peripheral blood mononuclear cells (PBMCs) were isolated by density gradient centrifugation using Ficoll-Hypaque (TBD Science) and washed twice with RPMI-1640/10% FBS (Life Technologies). All healthy controls (*n* = 12) were unexposed to known risk factors, displayed no symptoms of infection, and had no recent treatment or conditions that would interfere with inflammatory responses. All participants and healthy controls had been tested for SARS-CoV-2.

### RNA sequencing and data analysis

RNA was purified from PBMCs of the participants using the RNeasy Plus Mini Kit (Qiagen, Cat.74134). RNA concentration and integrity were determined with the RNA Bioanalyzer (Agilent, USA). Transcriptome sequencing was performed on the Illumina HiSeq 4000 platform (Illumina, United Kingdom). Reads with low quality or adaptor contamination were filtered out, and the remaining data were mapped to the human genome GRCh38 using HISAT2 v2.1.0 (15) with default parameters. Genes were quantified using the featureCounts program of the Subread package. Differential gene expression analysis of the expression quantification results was conducted using *R* packages, including edgeR, limma-voom, and glimma. Differentially expressed genes (DEGs) were determined with adjusted *p*-values ≤0.05 and absolute log fold-change ≥1. Gene set enrichment analysis (GSEA) was performed using the *R* package EGSEA. For the hospitalized cases, the compositions of immune cells were provided by the hospital using routine blood test. However, for the second- and third-generation cases, they were not hospitalized, and therefore, we did not have their data on the composition of immune cells from the hospital. To infer the composition of immune cells for these cases, raw gene counts were normalized as transcripts per million (TPM) and processed using the CIBERSORT algorithm v1.06 (16) with the original CIBERSORT gene signature file LM22 and 100 permutations.

### Cytokine and chemokine measurements

A multiplex-biometric immunoassay based on fluorescent microspheres conjugated with monoclonal antibodies specific for target cytokines [Bio-Plex ProTM Human Cytokine Array 27-Plex Group I and 21-Plex Group II Kits (Bio-Rad) on a Luminex200TM] was performed to assess plasma cytokine levels. Concentrations of 48-plex cytokines in plasma were examined. The data were analyzed using the Luminex data collection software (version 6.1) (17).

### Statistical analysis

Fisher’s exact test was used for categorical variables, and the Mann–Whitney test was used for continuous non-normally distributed variables. The unpaired, two-tailed *t*-test was used to determine differences in the cytokine and chemokine levels, with GraphPad Prism. A *p*-value between 0.01 and 0.05, between 0.001 and 0.01, and between 0.0001 and 0.001 was considered statistically significant, very significant, and extremely significant, respectively.

## Results

### Epidemiological and clinical characteristics

**Figure 2:**
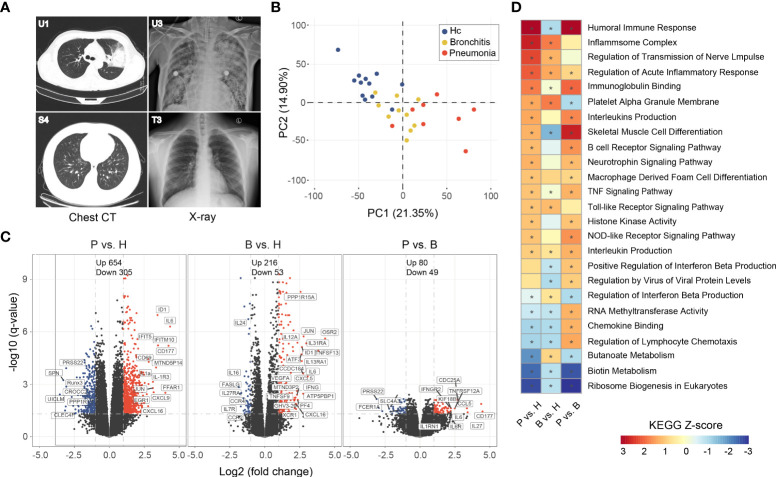
Imaging features of representative *C. psittaci* cases with pneumonia and gene expression analysis in PBMC of the *C. psittaci* cases compared to healthy controls. **(A)** Chest CTs obtained from U1 and S4, and X-ray obtained from U3 and T3. **(B)** PCA loading plot based on all DEGs. Autoscaling of the data was performed. **(C)** Volcano plot of DEGs comparing the pneumonia cases versus healthy (P vs. H), the bronchitis cases versus healthy (B vs. H), and the pneumonia cases versus the bronchitis cases (P vs. B). The names of DEGs related to immune response are shown. **(D)** Functional enrichment analysis of DEGs with the ingenuity pathway analysis. Asterisks (*) indicate *p*-values <0.05 and absolute *Z*-score ≥1.

PBMCs were collected from laboratory-confirmed *C. psittaci* cases from the Xintai Third People’s Hospital, China (**Figure 1** and **Table S1**), and the majority of cases had a clear history of exposure to *C. psittaci*. Based on clinical symptoms, these cases were further classified into two groups: bronchitis and pneumonia. The nine bronchitis cases (S1, S2, S4–S6, T1–T3, and U2) only presented with bronchitis and were not hospitalized, whereas the eight pneumonia cases (P1–P6, U1, and U3) showed pneumonia and pulmonary consolidation, and were hospitalized. The baseline and disease characteristics of the 17 *C. psittaci* cases are summarized in **Table 1**. The median age of these cases was 37.6 years, ranging from 2 to 65 years. The pneumonia cases showed respiratory distress on admission, accompanied by fever, headache, cough, and other symptoms. Routine testing showed that the proportion of neutrophils, erythrocyte sedimentation rate, and lactate dehydrogenase levels was elevated in most cases in the pneumonia group, and alanine aminotransferase and glutamic oxaloacetic transaminase also increased in some cases, suggesting hepatic damage (**Table S2**). Chest CT and X-ray examinations found that the pneumonia group had unilateral pulmonary high-attenuation consolidation and diffuse flaky high-density shadows, with enlargement of bilateral hila; the bronchitis group had pulmonary ground-glass opacity, tree-in-budding changes, and blurred edges (**Figure 2A**). Two of the pneumonia cases (P1 and P2) had recurrent hemoptysis. In contrast, the bronchitis group presented with cough or low-grade fever (**Table S1**).

**Table 1:** Clinical characteristics of the psittacosis groups and controls.

	Pneumonia cases (*n* = 8)	Bronchitis cases (*n* = 9)	Healthy controls (*n* = 12)
**Demographics**			
Age (years)	41.9 ± 17.1	33.8 ± 31.8	33.5 ± 10.5
Male/female	2/6	3/6	3/9
Smoking (%)	1 (12.5)	1 (11.1)	0
**Symptoms at admission (%)**			
Fever	8 (100)	3 (33.3)	0
Cough	8 (100)	7 (77.8)	0
Dyspnea	7 (87.5)	1 (11.1)	0
Headache	5 (62.5)	4 (44.4)	0
Myalgia	4 (50)	2 (22.2)	0
Nausea	4 (50)	0	0
Vomiting	4 (50)	0	0
Hemoptysis	2 (25)	0	0
Gatism	1 (12.5)	0	0
**Comorbidities (%)**			
Hypertension	2 (25)	2 (22.2)	1 (8.3)
Heart disease	1 (12.5)	1 (11.1)	0
Diabetes	0	1 (11.1)	0
COVID19	0	0	0
**Treatment (%)**			
Mechanical ventilation	4 (50)	0	/
Antibiotic therapy	8 (100)	8 (88.9)	/
Hormone therapy	4 (50)	0	/
**Outcome (%)**			
Recovery	7 (87.5)	9 (100)	/
Death	1 (12.5)	0	/

Data presented as mean ± SD or absolute number (percentage of group total)./: Not applicable.

### Transcriptomic sequencing and differentially expressed gene analysis

Eight pneumonia cases, nine bronchitis cases, and 12 healthy volunteers were included in the transcriptome analysis. A total of >37 million reads were generated by RNA-Seq, of which >94% could be mapped to the human genome (**Table S3**). DEGs were identified by comparing adjusted *p*-values (*p*-value <0.05) and fold change (FC) ratios (|log2FC|≥1) of the pneumonia, bronchitis, and healthy groups (**Table S4**). Principal component analysis (PCA) showed that the *C. psittaci* cases and healthy controls formed distinct clusters, indicating different characteristics of the DEGs; with a partial overlap between the pneumonia and the bronchitis clusters (**Figure 2B**).

Volcano plots showed that the number of DEGs in the pneumonia group was significantly higher than those in both the bronchitis and healthy control groups (e.g., the number of upregulated genes, 654 vs. 216 and 80, respectively), indicating that *C. psittaci* infection might severely interfere with transcriptome homeostasis in peripheral blood immune cells in more severe disease (**Figure 2C**). Analysis of expression changes in DEGs in the pneumonia and bronchitis cases (|log2FC| ≥ 2) showed that numerous genes were upregulated in the pneumonia cases, including interferon-stimulated genes (ISGs) (*IFITM3*, *IFITM10*, and *IFIT5*) involved in innate immune responses, and pro-inflammatory cytokines/chemokines (*CXCL1*, *CXCL9*, *IL-1a*, *CXCL16*, *CXCL3*, and *IL6R*), and *IL-1R3* genes that play multi-functional roles in inflammatory diseases (**Table S4**). In addition, we observed significant upregulation of *SOCS3* and *IL1RN*, both of which encode cytokine antagonists, indicating the possibility that negative feedback loops were induced. *EGR1* expression was also elevated in the pneumonia group, which inhibits pro-inflammatory genes in developing and mature macrophages. *CD69*, *JUN*, and *JUNB*, which function in immune cell proliferation and transformation, were also upregulated. However, *Runx3* and SPN, implicated in morphological development and migration of immune cells, were downregulated. *CD177*, involved in the bactericidal activity of neutrophils, was significantly elevated in all pneumonia cases but not bronchitis cases, indicating neutrophil activation, which may be beneficial to the recovery of psittacosis cases. A large number of DEGs were also identified when comparing bronchitis cases to healthy controls, albeit with much lower fold changes (**Table S4**).

### Functional enrichment analysis of the regulatory genes

*C. psittaci* infection can cause dynamic changes in gene expression in specific biological processes. We performed KEGG functional enrichment analysis on up- and downregulated genes in PBMCs (**Figure 2D**), and found that upregulated genes were mainly enriched in the pathways: “Inflammasome complex” (*Z*-score = 2.7) and “acute inflammatory response regulation”, indicating that a series of processes related to inflammatory responses were activated. Other pathways, including “Humoral immune response” (*Z*-score = 3.1), “Interleukins production”, “B cell receptor signaling pathway”, “TNF signaling pathway”, and “Macrophage derived foam cell differentiation”, were also activated, indicative of a higher level of immune activation in the pneumonia group. In addition, “Toll-like receptor signaling pathway” and “NOD-like receptor signaling pathway” involved in innate immunity were also activated. In fact, the most abundant biological processes also included “regulation of transmission of nerve impulse” and “Neurotrophin signaling pathway”. On the contrary, the downregulation of genes in PMBCs of cases were related to biological processes, including “Ribosome biogenesis in eukaryotes” (*Z*-score = −2.8), “Biotin metabolism”, and “Butanoate metabolism”. Therefore, functional analysis also revealed that psittacosis results in strong immune responses and hypercytokinemia in the pneumonia group.

### Cytokine expression profiling

**Figure 3:**
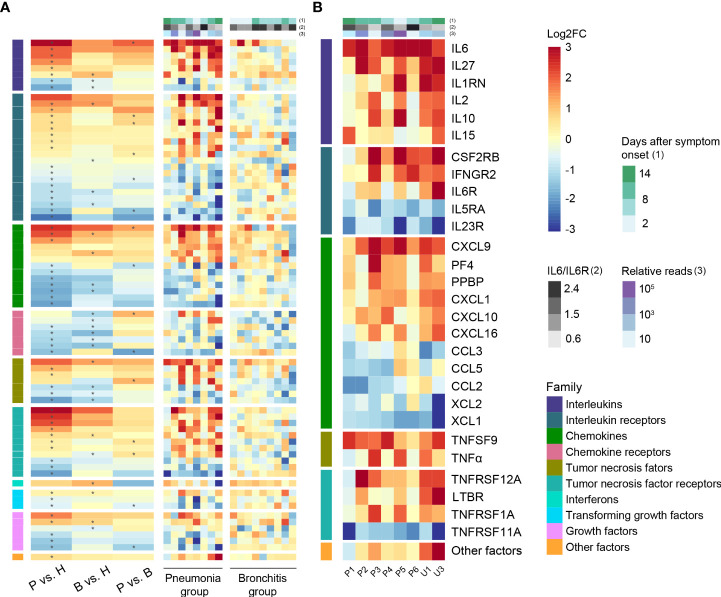
Cytokine-related gene expression in *C. psittaci* cases. **(A)** Heatmap of DEGs encoding cytokines and chemokines. **(B)** Heatmap of representative DEGs encoding cytokines and chemokines in PBMC of the pneumonia case samples (*n* = 8). Asterisks (*) indicate significant DEGs (absolute log2FC ≥ 1, *p*-value < 0.05). The ratios of IL6 to IL6R and relative reads are shown in **(A, B)**.

In order to understand whether the concentration of various cytokines/chemokines post-*C. psittaci* infection was closely correlated with disease progression, we profiled the DEGs in PBMCs of the pneumonia and bronchitis groups, which were divided into 10 classes with statistically significant deregulated genes marked with asterisks (**Figures 3A, B**). A number of genes, including “Interleukins”, “Chemokines”, and “Tumor necrosis factor receptors”, were the most significantly upregulated in the pneumonia cases. Notably, the vascular endothelial marker *IL-6* was significantly upregulated in the eight pneumonia cases, and *IL-27*, which negatively regulates inflammation, was also activated, suggesting that IL-6 may be an important marker of disease severity of psittacosis. The T-lymphocyte chemokine *CXCL9* was the most upregulated DEG. The upregulation of *CXCL9* was unique to the pneumonia cases, indicating that *CXCL9* is involved in the pathogenesis of psittacosis. Increased transcription of chemokine receptors CCR2 (*CCL2/monocyte chemoattractant protein-1 (MCP-1)* receptor) and *CCR5* (*CCL3/MIP-1A* receptor) were observed (**Table S4**), indicating that these inflammatory signals were activated, but receptors *XCR1*, *CCR4*, and *ACKR3* were downregulated in the pneumonia group. In addition, we also observed high levels of the macrophage chemokines *CXCL10/interferon-inducible protein-10 (IP-10)* and *CCL2/MCP-1*, as well as the neutrophil chemokine *CXCL8* (*IL-8*), which mobilize and recruit immune cells in the bone marrow. Compared with the bronchitis cases, the elevated expression of these chemokines may contribute to hypercytokinemia in the pneumonia cases. Taken together, our results revealed a unique profile of interleukin and chemokine responses in psittacosis cases hospitalized with pneumonia.

### Plasma inflammatory cytokine responses

**Figure 4:**
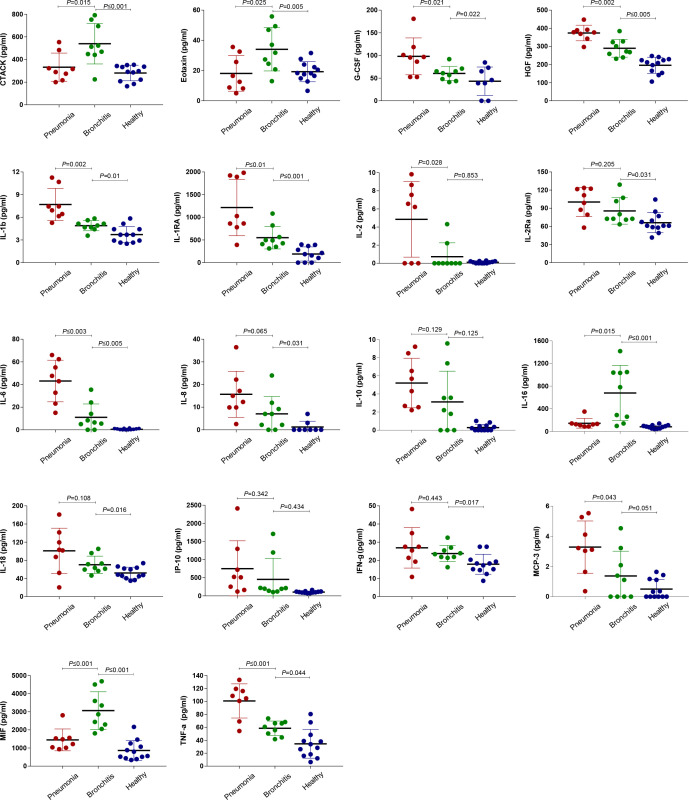
Comparison of plasma cytokine/chemokine concentrations between healthy controls as well as the pneumonia and bronchitis cases infected with *C. psittaci*. Samples were collected at the earliest time point for assays measuring the concentrations of 48 cytokines and chemokines. Values are presented in pg/ml. The mean ± standard error is shown for each group and the *p*-values are determined by *t*-test. A *p*-value between 0.01 and 0.05, between 0.001 and 0.01, and between 0.0001 and 0.001 was considered statistically significant, very significant, and extremely significant, respectively.

To further validate the correlation between clinical symptoms of psittacosis and the concentrations of inflammatory cytokines, we tested 48 plasma cytokines in the bronchitis and pneumonia groups. Elevated concentrations of both pro- and anti-inflammatory plasma cytokines were observed in the pneumonia group (**Figure 4** and **Table S5**), including G-CSF, HGF, IL-1b, IL-1RA, IL-2, IL-2Ra, IL-6, IL-10, IL-18, IP-10, monocyte chemoattractant protein-3 (MCP-3), and TNF-α. In particular, plasma IL-6 was significantly elevated and increased approximately 30-fold in pneumonia cases, with an average value of 43.11 pg/ml. We also noted the elevated expression level of IL6R in lymphocytes, suggesting that the IL-6/IL6R axis may be involved in psittacosis pathophysiology. IL1RN and IL1b were also significantly upregulated interleukin genes in pneumonia cases (**Figure 3B** and **Table S4**), which were consistent with the increased expression of plasma IL-1Ra and IL-1b proteins (**Figure 4** and **Table S5**). The secretion of TNF-α was also drastically increased, with the average concentration of TNF-α reaching 101.3 pg/ml. In contrast, during the recovery period of the pneumonia group, such as for cases P1, P2, and U1, there were decreases in certain cytokines, such as IL-2, IL-6, MCP-3, and TNF-α (**Table S5**). These results suggested potential psittacosis-mediated inflammatory mediator changes, consistent with the important role of inflammatory monocytes/macrophages in the immunopathogenesis of severe psittacosis. Of particular concern was the significant elevation of plasma D-dimer and C-reactive protein (CRP) in each of the eight pneumonia cases (**Table S2**), which correlated with an increased risk of a persisting cytokine storm. As shown in the heatmap, one case (P5) with the highest *C. psittaci* reads (indicated by the number of reads per million: 710.9) (**Table S6**) exhibited upregulated expression of interleukins and TNF (**Figures 3A, B**), suggesting that higher *C. psittaci* burden may lead to stronger pro-inflammatory responses. In the fatal case (U3), the cytokines showed pronounced abnormal expression compared with other pneumonia cases (**Figure 3B**). However, the levels of CTACK, Eotaxin, IL-16, macrophage migration inhibitory factor (MIF), and MCP-1 in the pneumonia group were significantly lower than those in the bronchitis group (**Figure 4** and **Table S5**). These results indicate that the combined action of a variety of inflammatory cytokines induces uncontrolled inflammatory responses, leading to severe disease.

### Cell component analysis

**Figure 5:**
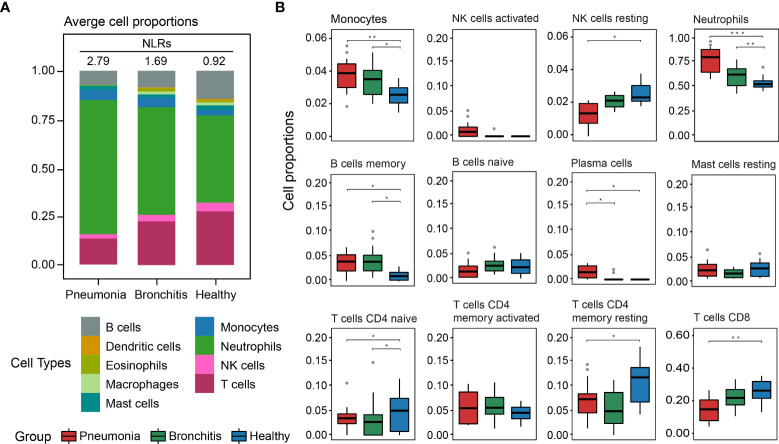
Composition of immune cells in PBMCs in *C. psittaci* cases compared to healthy controls determined from transcriptome data. **(A)** The proportion of nine major immune cell types. The neutrophil-to-lymphocyte ratios (NLRs) are shown in **(A)**. **(B)** The proportion of 12 immunity-related cell subtypes. Asterisks represent significant differences between groups (*p-value < 0.05, **p-value < 0.01, ***p-value < 0.001, Mann–Whitney test).

Based on the transcriptome data, we evaluated the proportions of immune cells in PBMCs through CIBERSORT. The results showed that neutrophils and T lymphocytes had the highest percentages of cellular components in the healthy controls (**Figure 5A**). This suggested the accuracy of the PBMCs sequencing and the effectiveness of our analytical approach. However, ratios of neutrophils in PBMCs in the pneumonia group increased more significantly than in the bronchitis group, with an average value of 77.5%. In addition, the ratios of T lymphocytes, B lymphocytes, macrophages and dendritic cells in the pneumonia cases were lower than those in the bronchitis group (**Figure 5B**). Notably, the neutrophil-to-lymphocyte ratio (NLR) of the pneumonia group was higher than that of the bronchitis group (2.79 vs. 1.69). In particular, the highest NLR of 7.72 was observed in the fatal case—U3. This suggested that the higher the NLR, the more severe the condition or the greater risk of poor prognosis in patients with psittacosis.

## Discussion

Transcriptomic analyses upon *C. psittaci* infection are extremely useful to identify differential gene expression and functional gene classification (18, 19). In particular, it has been reported that IFN-γ-induced *C. psittaci* persistence in HeLa cells resulted in the upregulation of 68 and downregulation of 109 genes after persistent psittacosis infection (18). Comparisons of transcriptomes also indicated considerable differences in expression of homologous virulence factors that may contribute to the differing infection rates and disease outcomes following *C. psittaci* infection (19). Recently, there are increasingly frequent reports of severe and fatal cases of psittacosis (4, 5); however, because the vast majority of psittacosis are individual cases, there are few studies on immune responses of psittacosis based on numerous laboratory-confirmed infections. In December 2020, a large-scale human-to-human transmission of *C. psittaci* was reported in Shandong, China, causing nosocomial and within-family infections, with several cases rapidly progressing from cold-like symptoms to pneumonia and death. Herein, through transcriptome and cytokine analysis of PBMCs in confirmed psittacosis cases with heterogeneous symptoms and healthy controls, gene expression and inflammatory cytokine profiles were obtained, which directly reflect the heterogeneous host immune response against *C. psittaci* infection, providing an invaluable opportunity to understand psittacosis pathogenesis.

When *C. psittaci* invades human or animal cells or tissues, a variety of inflammatory cytokines are upregulated, particularly IL-6, IL-8, MCP-1, GM-CSF, TNF-α, and anti-inflammatory factor IL-10 (20–22), and the TLR4/Mal/MyD88/NF-κB signaling axis is activated, which contributes to the innate immune responses (23), which may play different pathogenic roles in the process of *C. psittaci* infection. Consistent with the previous findings, we found that levels of a variety of cytokines, such as IL-6, IL-1RN, and TNF-α, increased rapidly in the pneumonia cases, thereby accelerating the inflammatory response (24). The inflammatory response promotes abnormal liver function and leads to the production of CRP and D-dimer, which have also been identified as biomarkers of disease severity in SARS-CoV-2 and avian influenza (25–27). Strikingly, this was observed in all of our eight psittacosis cases with pneumonia. Elevated CRP and D-dimer activate the complement system recruiting inflammatory cells and increasing vascular permeability, leading to disease deterioration (28). In addition, we found that *C. psittaci* also induced the excessive expression of cytokine IL-10. Similar to high IL-6 and IL-10 activity found in COVID-19 patients (29), elevated IL-10 plays a key role in the development of pulmonary injury and fibrosis. Persistent pulmonary damage prompted a further increase in the release of lactate dehydrogenase into the plasma of the pneumonia cases, and the lactate dehydrogenase reached 1231 U/L in the fatal case. This is in agreement with the observation that the psittacosis with pneumonia showed unilateral ground glass opacities and pulmonary invasive lesions upon admission. Taken together, these results suggest that continuous excessive release of a variety of inflammatory factors at psittacosis onset is closely correlated with the occurrence and progression to pneumonia and pulmonary injury.

Chemokines are essential to neutrophil recruitment and accumulation (30–32). We found significant upregulated expression of multiple chemokines and receptors (*CXCL1*, *CXCL9*, and *CXCL10/IP-10*) in severe psittacosis cases. Consistently, several chemoattractants of monocytes and other immune cells were also upregulated. In patients with acute respiratory distress syndrome caused by bacterial infection (*Serratia* and *Enterobacter*), the alveolar cavity was occupied by infiltrating neutrophils and monocytes (33), indicating the pathogenic role of these immune cells. Therefore, our study reveals a possible chemokine-dominant cytokine response in psittacosis with pneumonia.

Chemokines and their receptors play important roles in cytokine migration and immune cell activation at the site of infection (34). The chemokine receptors *CCR2* (*CCL2/MCP-1* receptor) and *CCR5* (*CCL3/MIP-1A* receptor) in deficient mice exhibit defects in directing inflammatory cells to airways after infection (35). Our data revealed increased CCR2 and CCR5, indicating activation of inflammatory signals. However, the general downregulation of expression of *CCL2*, *CCL3* and *CCL5* in the pneumonia cases may be related to decreases in numbers of peripheral T and B lymphocytes. We conclude that *C. psittaci* infection induces high-level expression of a variety of chemokines, which promote the rapid proliferation of neutrophils and facilitate the migration to the infection sites within the lung tissue, consistent with the monocyte and lymphocyte infiltrates in the lung tissues of *C. psittaci* patients (36).

Our study has some limitations. First, we only collected cross-sectional samples of the cases with different conditions (pneumonia or bronchitis), and longitudinal samples were not obtained. Therefore, our results cannot resolve the kinetics of the inflammatory markers, especially those occurring during the recovery period. Second, changes in BALF may reflect the inflammatory conditions prevailing in lung more directly, but we only obtained BALF from the five pneumonia patients (P1, P2, P4, P5, and U3). Third, the clinical samples that we collected were not timely enough and most patients had already been treated with antibiotics (doxycycline) when sampling, which may affect the inflammatory/immune responses to *C. psittaci*. For example, tetracycline minocyclines can inhibit the proliferation of T cells and the production of the cytokines IL-2, IFN-γ, IFN-α, and TNF-α by chelating Ca^2+^ (37). The clinically preferred drug for the treatment of psittacosis is doxycycline, which may have overlapping functions and mechanisms similar to tetracycline.

In summary, we collected blood samples from eight pneumonia and nine bronchitis cases with psittacosis during an unprecedented epidemic in China, identified several plasma biomarkers distinct to the pneumonia cases, and revealed that the hypercytokinemia caused by the combined action of a variety of cytokines led to strong inflammatory responses. These findings deepen our understanding of the heterogeneous immunological responses that occur during psittacosis and should prove helpful for developing both preventative and therapeutic strategies.

## Data availability statement

The datasets presented in this study can be found in online repositories. The name of the repository and accession number can be found below: NCBI Gene Expression Omnibus; GSE202947.

## Ethics statement

Written informed consent was obtained from the individual(s), and minor(s)’ legal guardian/next of kin, for the publication of any potentially identifiable images or data included in this article.

## Author contributions

WS and LM designed and supervised this study. ZZ, CM, JW, WL, LW, HC, and HG collected the samples and ZZ, PW, and CQ performed the experiments. ZZ, PW, and CY performed statistical analysis. WS and ZZ wrote the manuscript. WS and MC edited the paper. All authors contributed to the article and approved the submitted version.

## Funding

This work was supported by the Academic Promotion Programme of Shandong First Medical University [grant number 2019QL006], the Natural Science Foundation of Shandong Province [grant number ZR2021MC001], and the Medical and Health Science and Technology Development Program of Shandong Province [grant number 202001060452].

## Conflict of interest

The authors declare that the research was conducted in the absence of any commercial or financial relationships that could be construed as a potential conflict of interest.

## Publisher’s note

All claims expressed in this article are solely those of the authors and do not necessarily represent those of their affiliated organizations, or those of the publisher, the editors and the reviewers. Any product that may be evaluated in this article, or claim that may be made by its manufacturer, is not guaranteed or endorsed by the publisher.

## References

[1] Smith KA Bradley KK Stobierski MG Tengelsen LA National Association of State Public Health Veterinarians Psittacosis Compendium C. Compendium of measures to control *Chlamydophila psittaci* (formerly *Chlamydia psittaci*) infection among humans (psittacosis) and pet birds, 2005. J Am Vet Med Assoc (2005) 226(4):532–9. doi: 10.2460/javma.2005.226.532 15742693

[2] Li N Li S Tan W Wang H Xu H Wang D. Metagenomic next-generation sequencing in the family outbreak of psittacosis: the first reported family outbreak of psittacosis in China under COVID-19. Emerg Microbes Infect (2021) 10(1):1418–28. doi: 10.1080/22221751.2021.1948358 PMC8284143 34176434

[3] Katsura D Tsuji S Kimura F Tanaka T Eguchi Y Murakami T. Gestational psittacosis: A case report and literature review. J Obstet Gynaecol Res (2020) 46(5):673–7. doi: 10.1111/jog.14217 32077210

[4] Shaw KA Szablewski CM Kellner S Kornegay L Bair P Brennan S. Psittacosis outbreak among workers at chicken slaughter plants, Virginia and Georgia, USA, 2018. Emerg Infect Dis (2019) 25(11):2143–5. doi: 10.3201/eid2511.190703 PMC6810211 31625859

[5] Gu L Liu W Ru M Lin J Yu G Ye J. The application of metagenomic next-generation sequencing in diagnosing *Chlamydia psittaci* pneumonia: a report of five cases. BMC Pulm Med (2020) 20(1):65. doi: 10.1186/s12890-020-1098-x 32178660 PMC7077129

[6] Stewardson AJ Grayson ML. Psittacosis. Infect Dis Clin North Am (2010) 24(1):7–25. doi: 10.1016/j.idc.2009.10.003 20171542

[7] Yung AP Grayson ML. Psittacosis–a review of 135 cases. Med J Aust (1988) 148(5):228–33. doi: 10.5694/j.1326-5377.1988.tb99430.x 3343952

[8] Knittler MR Sachse K. *Chlamydia psittaci*: update on an underestimated zoonotic agent. Pathog Dis (2015) 73(1):1–15. doi: 10.1093/femspd/ftu007 25853998

[9] Radomski N Einenkel R Müller A Knittler MR. Chlamydia-host cell interaction not only from a bird's eye view: some lessons from chlamydia psittaci. FEBS Lett (2016) 590(21):3920–40. doi: 10.1002/1873-3468.12295 27397851

[10] Beeckman DS Vanrompay DC. Zoonotic *Chlamydophila psittaci* infections from a clinical perspective. Clin Microbiol Infect (2009) 15(1):11–7. doi: 10.1111/j.1469-0691.2008.02669.x 19220335

[11] Huang KJ Su IJ Theron M Wu YC Lai SK Liu CC. An interferon-gamma-related cytokine storm in SARS patients. J Med Virol (2005) 75(2):185–94. doi: 10.1002/jmv.20255 PMC7166886 15602737

[12] de Jong MD Simmons CP Thanh TT Hien VM Smith GJ Chau TN. Fatal outcome of human influenza a (H5N1) is associated with high viral load and hypercytokinemia. Nat Med (2006) 12(10):1203–7. doi: 10.1038/nm1477 PMC4333202 16964257

[13] Huang C Wang Y Li X Ren L Zhao J Hu Y. Clinical features of patients infected with 2019 novel coronavirus in wuhan, China. Lancet (2020) 395(10223):497–506. doi: 10.1016/S0140-6736(20)30183-5 PMC7159299 31986264

[14] Zhang Z Zhou H Cao H Ji J Zhang R Li W. Human-to-human transmission of *Chlamydia psittaci* in China, 2020: an epidemiological and etiological investigation. Lancet Microbe (2022) 3(7):e512ߝ20. doi: 10.1016/S2666-5247(22)00064-7 35617977

[15] Kim D Paggi JM Park C Bennett C Salzberg SL. Graph-based genome alignment and genotyping with HISAT2 and HISAT-genotype. Nat Biotechnol (2019) 37(8):907–15. doi: 10.1038/s41587-019-0201-4 PMC7605509 31375807

[16] Newman AM Liu CL Green MR Gentles AJ Feng W Xu Y. Robust enumeration of cell subsets from tissue expression profiles. Nat Methods (2015) 12(5):453–7. doi: 10.1038/nmeth.3337 PMC4739640 25822800

[17] Guo J Guo X Wang Y Tian F Luo W Zou Y. Cytokine response to hantaan virus infection in patients with hemorrhagic fever with renal syndrome. J Med Virol (2017) 89(7):1139–45. doi: 10.1002/jmv.24752 27943332

[18] Chen Y Wang C Mi J Zhou Z Wang J Tang M. Characterization and comparison of differentially expressed genes involved in *Chlamydia psittaci* persistent infection *in vitro* and *in vivo* . Vet Microbiol (2021) 255:108960. doi: 10.1016/j.vetmic.2020.108960 33667981

[19] Beder T Saluz HP. Virulence-related comparative transcriptomics of infectious and non-infectious chlamydial particles. BMC Genomics (2018) 19(1):575. doi: 10.1186/s12864-018-4961-x 30068313 PMC6090853

[20] Li Q Li X Quan H Wang Y Qu G Shen Z. IL-10^-/-^ enhances DCs immunity against *Chlamydia psittaci* infection *via* OX40L/NLRP3 and IDO/Treg pathways. Front Immunol (2021) 12:645653. doi: 10.3389/fimmu.2021.645653 34093535 PMC8176032

[21] Buxton D Anderson IE Longbottom D Livingstone M Wattegedera S Entrican G. Ovine chlamydial abortion: characterization of the inflammatory immune response in placental tissues. J Comp Pathol (2002) 127(2-3):133–41. doi: 10.1053/jcpa.2002.0573 12354524

[22] Rasmussen SJ Eckmann L Quayle AJ Shen L Zhang YX Anderson DJ. Secretion of proinflammatory cytokines by epithelial cells in response to *Chlamydia infection* suggests a central role for epithelial cells in chlamydial pathogenesis. J Clin Invest (1997) 99(1):77–87. doi: 10.1172/JCI119136 PMC507770 9011579

[23] Chen Q Li Y Yan X Sun Z Wang C Liu S. *Chlamydia psittaci* plasmid-encoded CPSIT_P7 elicits inflammatory response in human monocytes *via* TLR4/Mal/MyD88/NF-κB signaling pathway. Front Microbiol (2020) 11:578009. doi: 10.3389/fmicb.2020.578009 33343522 PMC7744487

[24] Zhou Y Fu B Zheng X Wang D Wei H. Aberrant pathogenic GM-CSF ^+^ T cells and inflammatory CD14 ^+^ CD16 ^+^ monocytes in severe pulmonary syndrome patients of a new coronavirus. bioRxiv (2020):1ߝ6. doi: 10.1101/2020.02.12.945576

[25] Chen N Zhou M Dong X Qu J Gong F Han Y. Epidemiological and clinical characteristics of 99 cases of 2019 novel coronavirus pneumonia in wuhan, China: a descriptive study. Lancet (2020) 395(10223):507–13. doi: 10.1016/S0140-6736(20)30211-7 PMC7135076 32007143

[26] Huang Y Zhou H Yang R Xu Y Feng X Gong P. Clinical characteristics of 36 non-survivors with COVID-19 in wuhan, China. medRxiv (2020). doi: 10.1101/2020.02.27.20029009

[27] Wang ZF Su F Lin XJ Dai B Kong LF Zhao HW. Serum d-dimer changes and prognostic implication in 2009 novel influenza A(H1N1). Thromb Res (2011) 127(3):198–201. doi: 10.1016/j.thromres.2010.11.032 21216444

[28] Shen B Yi X Sun Y Bi X Du J Zhang C. Proteomic and metabolomic characterization of COVID-19 patient sera. Cell (2020) 182(1):59–72.e15. doi:10.1016/j.cell.2020.05.032 PMC7254001 32492406

[29] Han H Ma Q Li C Liu R Zhao L Wang W. Profiling serum cytokines in COVID-19 patients reveals IL-6 and IL-10 are disease severity predictors. Emerg Microbes Infect (2020) 9(1):1123–30. doi: 10.1080/22221751.2020.1770129 PMC7473317 32475230

[30] Donnelly SC Strieter RM Kunkel SL Walz A Robertson CR Carter DC. Interleukin-8 and development of adult respiratory distress syndrome in at-risk patient groups. Lancet (1993) 341(8846):643–7. doi: 10.1016/0140-6736(93)90416-E 8095568

[31] Frevert CW Huang S Danaee H Paulauskis JD Kobzik L. Functional characterization of the rat chemokine KC and its importance in neutrophil recruitment in a rat model of pulmonary inflammation. J Immunol (1995) 154(1):335–44. 7995953

[32] Miller EJ Cohen AB Nagao S Griffith D Maunder RJ Martin TR. Elevated levels of NAP-1/interleukin-8 are present in the airspaces of patients with the adult respiratory distress syndrome and are associated with increased mortality. Am Rev Respir Dis (1992) 146(2):427–32. doi: 10.1164/ajrccm/146.2.427 1489135

[33] Matute-Bello G Frevert CW Martin TR. Animal models of acute lung injury. Am J Physiol Lung Cell Mol Physiol (2008) 295(3):L379–L99. doi: 10.1152/ajplung.00010.2008 PMC2536793 18621912

[34] Griffith JW Sokol CL Luster AD. Chemokines and chemokine receptors: positioning cells for host defense and immunity. Annu Rev Immunol (2014) 32:659–702. doi: 10.1146/annurev-immunol-032713-120145 24655300

[35] Sheahan T Morrison TE Funkhouser W Uematsu S Akira S Baric RS. MyD88 is required for protection from lethal infection with a mouse-adapted SARS-CoV. PloS Pathog (2008) 4(12):e1000240. doi: 10.1371/journal.ppat.1000240 19079579 PMC2587915

[36] Hayashi Y Kato M Ito G Yamamoto K Kuroki H Matsuura T. [A case report of psittacosis and chlamydial isolation from a dead pet bird]. Nihon Kyobu Shikkan Gakkai Zasshi (1990) 28(3):535–40. 2214397

[37] Kloppenburg M Verweij CL Miltenburg AM Verhoeven AJ Daha MR Dijkmans BA. The influence of tetracyclines on T cell activation. Clin Exp Immunol (1995) 102(3):635–41. doi: 10.1111/j.1365-2249.1995.tb03864.x PMC1553388 8536384

