# Supplementary material for: Host inflammatory response is the major factor in the progression of Chlamydia psittaci pneumonia
Source: Front Immunol. 2022 Sep 2;13:929213. doi: 10.3389/fimmu.2022.929213 (PMC9478202; doi:10.3389/fimmu.2022.929213)
Supplement: Supplementary file 3 [file Table_3.docx]

**Table S3. The number and proportion of human genome reads identified in *C. psittaci* cases and healthy controls**

| Samples | Total reads number | Mapped reads number | Mapped to the human genome (%) |
| --- | --- | --- | --- |
| P1 | 40972366 | 39137311 | 95.52 |
| P2 | 37194618 | 35441084 | 95.29 |
| P3 | 37120452 | 35299291 | 95.09 |
| P4 | 37699768 | 35853477 | 95.10 |
| P5 | 40777642 | 38930355 | 95.47 |
| P6 | 43387552 | 41438000 | 95.51 |
| U1 | 40730160 | 38938775 | 95.60 |
| U3 | 43105602 | 41323349 | 95.87 |
| S1 | 41867664 | 39863783 | 95.21 |
| S2 | 38225612 | 36373661 | 95.16 |
| S4 | 42210536 | 40188949 | 95.21 |
| S5 | 40935540 | 39035511 | 95.36 |
| S6 | 39492462 | 37663856 | 95.37 |
| T1 | 45606722 | 43538600 | 95.47 |
| T2 | 46902260 | 44655520 | 95.21 |
| T3 | 38108216 | 36442036 | 95.63 |
| U2 | 38726034 | 36970118 | 95.47 |
| H1 | 41366290 | 39206676 | 94.78 |
| H2 | 42110592 | 39975846 | 94.93 |
| H3 | 39587642 | 37599045 | 94.98 |
| H4 | 37842818 | 35980861 | 95.08 |
| H5 | 46759422 | 43863282 | 93.81 |
| H6 | 50354718 | 47794330 | 94.92 |
| H7 | 50080466 | 47573181 | 94.99 |
| H8 | 44553020 | 42376578 | 95.11 |
| H9 | 46746146 | 44528380 | 95.26 |
| H10 | 46556976 | 44384882 | 95.33 |
| H11 | 39076258 | 37194014 | 95.18 |
| H12 | 44442876 | 42275793 | 95.12 |

In each case, the number of total reads (Total reads number) and the percent of reads that can be directly mapped to the reference genome of human (GRCh38) are presented. The mapping software is HISAT2 v2.1.0.
