# Supplementary material for: Host inflammatory response is the major factor in the progression of Chlamydia psittaci pneumonia
Source: Front Immunol. 2022 Sep 2;13:929213. doi: 10.3389/fimmu.2022.929213 (PMC9478202; doi:10.3389/fimmu.2022.929213)
Supplement: Supplementary file 6 [file Table_6.docx]

**Table S6. Next generation sequencing and identification of *C. psittaci* from P1-P6 and U1**

| **Patient ID** | **SampleType** | **SeqType** | **SeqData** | **Clean SeqData** | **Chromosome (NC_015470.1)** | | | **Plasmid (NC_015217.1)** | | | ***ompA* gene (X56980.1)** | | |
| --- | --- | --- | --- | --- | --- | --- | --- | --- | --- | --- | --- | --- | --- |
|  |  |  |  |  | **numreads** | **covbases (bp)** | **coverage (%)** | **numreads** | **covbases (bp)** | **coverage (%)** | **numreads** | **covbases (bp)** | **coverage (%)** |
| **P1** | Sputum | Metagenomics | 112,531,606 | 106,054,324 | 0 | 0 | 0 | 0 | 0 | 0 | 0 | 0 | 0 |
|  | BALF | Metagenomics | 131,584,594 | 123,703,568 | 16 | 889 | 0.08 | 0 | 0 | 0 | 0 | 0 | 0 |
|  | BALF | Metatranscriptomics | 93,565,518 | 90,341,526 | 8 | 568 | 0.05 | 0 | 0 | 0 | 0 | 0 | 0 |
| **P2** | Sputum | Metagenomics | 216,280,232 | 206,975,616 | 10 | 680 | 0.06 | 0 | 0 | 0 | 0 | 0 | 0 |
|  | BALF | Metagenomics | 126,562,072 | 118,769,608 | 6 | 276 | 0.02 | 0 | 0 | 0 | 0 | 0 | 0 |
|  | BALF | Metatranscriptomics | 90,774,236 | 87,482,078 | 32 | 1,753 | 0.15 | 0 | 0 | 0 | 0 | 0 | 0 |
| **P3** | Sputum | Metagenomics | 86,477,638 | 74,370,586 | 0 | 0 | 0 | 0 | 0 | 0 | 0 | 0 | 0 |
|  | Sputum | Metatranscriptomics | 8,016,122 | 7,503,656 | 12 | 931 | 0.08 | 0 | 0 | 0 | 0 | 0 | 0 |
| **P4** | Sputum | Metagenomics | 72,895,850 | 60,696,204 | 56 | 4,518 | 0.39 | 0 | 0 | 0 | 0 | 0 | 0 |
|  | BALF | Metagenomics | 224,745,206 | 212,474,352 | 680 | 45,409 | 3.88 | 8 | 483 | 6.39 | 2 | 77 | 4.64 |
|  | BALF | Metatranscriptomics | 84,502,442 | 79,082,470 | 26 | 1,298 | 0.11 | 0 | 0 | 0 | 0 | 0 | 0 |
| **P5** | Sputum | Metagenomics | 196,718,224 | 186,782,756 | 13,758 | 699,384 | 59.69 | 122 | 5797 | 76.75 | 20 | 1123 | 67.65 |
|  | Sputum | Metatranscriptomics | 112,978,480 | 109,798,384 | 61,272 | 97,668 | 8.34 | 6 | 567 | 7.51 | 160 | 1502 | 90.48 |
|  | BALF | Metagenomics | 115,289,846 | 107,952,952 | 1,536 | 122,243 | 10.43 | 24 | 1477 | 19.56 | 4 | 396 | 23.86 |
|  | BALF | Metatranscriptomics | 105,396,834 | 101,426,728 | 72,104 | 100,908 | 8.61 | 4 | 187 | 2.48 | 196 | 1266 | 76.27 |
| **P6** | Sputum | Metagenomics | 100,464,788 | 96,616,922 | 8 | 646 | 0.06 | 0 | 0 | 0 | 0 | 0 | 0 |
|  | Sputum | Metatranscriptomics | 106,122,742 | 101,844,650 | 8 | 383 | 0.03 | 0 | 0 | 0 | 0 | 0 | 0 |
| **U1** | Sputum | Metagenomics | 94,670,358 | 86,247,866 | 10 | 757 | 0.06 | 0 | 0 | 0 | 0 | 0 | 0 |
|  | Sputum | Metatranscriptomics | 93,614,208 | 90,590,502 | 284 | 4,536 | 0.39 | 0 | 0 | 0 | 0 | 0 | 0 |
